# Diphtheria and Anti-Toxin

**Published:** 1897-06-19

**Authors:** 


### Editor's Letter-Box.

[Our Correspondents are reminded that prolixity is a great bar to publication, and that brevity of style and conciseness of statement greatly facilitate early insertion.]

### DIPHTHERIA AND ANTI-TOXIN.

Mr. Lennox Browne writes; My attention has only just been drawn to a review on "Diphtheria and Anti-toxin," published in your issue of May 8th last. In it you say "great stress has been laid by Mr. Lennox Browne on the occurrence of albuminuria as a result of anti-toxin, but Dr. Tirard adds the weight of his own experience to the already large array of counter evidence." In reply to this I would draw your attention to the report on the use of anti-toxic serum in the treatment of diphtheria in the hospitals of the Metropolitan Asylums Board for 1896, wherein it is shown (Table XVI.) that albuminuria occurred in 60 per cent. of the 2,764 cases treated with anti-toxin, as against 40 per cent. on 1,412 cases not so treated (p. 9), the average—before anti-toxin was introduced—of this complication on 1,0C0 cases, in all of which analysis was made, having been in accord with these figures. I have only to add that, on the face of the report, all other complications and sequelæ are proved to be more frequent in the serum than in the non-serum treated cases, and by no manipulation of figures, or saving clauses, can this fact be gainsaid.

